# Number of dentists in the neighborhood and incidence of dental caries in the children permanent dentition

**DOI:** 10.1590/0103-6440202204321

**Published:** 2022-08-26

**Authors:** Marina Dutra Cósta, Bruna Brondani, Jessica Klöckner Knorst, Fausto Medeiros Mendes, Thiago Machado Ardenghi

**Affiliations:** 1 Federal University of Santa Maria(UFSM), Rio Grande do Sul(RS), Brazil.; 2 University of São Paulo(USP), São Paulo(SP), Brazil.

**Keywords:** Child, dental care, dental caries, longitudinal studies, public dental health

## Abstract

This study aimed to evaluate the influence of the number of dentists in the neighborhood on the incidence of dental caries in the children permanent dentition. This cohort began in 2010 (T1) with a random sample of 639 children (1 to 5 years-old) followed for 7 years, in southern Brazil. The follow-up reassessment (T2) took place in 2017. Untreated dental caries was evaluated at T2 through the Decayed, Missing, and Filled surfaces index (DMF-S). The number of dentists in the neighborhood was obtained from the city’s official database and used as a contextual variable. Socioeconomic, demographic, and oral health variables at the individual level were evaluated at T1. A multilevel Poisson regression was performed to evaluate the influence of the predictor variables in the incidence of untreated dental caries. From 639 children at T1, 449 were reassessed at T2 (a 70.3% retention rate). The mean of decayed surfaces at T2 was 0.92 (SE 0.01). The greater the number of dentists in the neighborhoods where the children lived, the lower the incidence of dental caries. Children with low socioeconomic status, who have not routinely visited the dentist in the last 6 months, who presented a experience of dental caries, and whose parents perceived their oral health as fair/poor showed a higher incidence of surfaces with untreated dental caries. As conclusion, children who live in neighborhoods with fewer dentists have a higher incidence of untreated dental caries in permanent dentition.

## Resumo

Este estudo teve como objetivo avaliar a influência do número de cirurgiões-dentistas do bairro na incidência de cárie dentária na dentição permanente. Esta coorte teve início em 2010 (T1) com uma amostra aleatória de 639 crianças (1 a 5 anos) acompanhadas por 7 anos, no sul do Brasil. A reavaliação (T2) ocorreu em 2017. A cárie dentária não tratada foi avaliada no T2 por meio do índice de superfícies cariadas, perdidas e obturadas (CPO-S). O número de dentistas do bairro foi obtido no banco de dados oficial da cidade e usada como uma variável contextual. Variáveis socioeconômicas, demográficas e de saúde bucal no nível individual foram avaliadas no T1. Foi realizada uma análise de regressão de Poisson multinível para avaliar a influência das variáveis preditoras na incidência de cárie dentária não tratada. Das 639 crianças no T1, 449 foram reavaliadas no T2 (taxa de retenção de 70,3%). A média das superfícies cariadas no T2 foi de 0,92 (EP 0,01). Quanto maior o número de dentistas nos bairros em que as crianças residiam, menor era a incidência de cárie dentária. Crianças com baixo nível socioeconômico, que não consultaram rotineiramente o dentista nos últimos 6 meses, que apresentaram experiência de cárie dentária e cujos pais perceberam sua saúde bucal como regular/ruim apresentaram maior incidência de superfícies com cárie dentária não tratada. Como conclusão, crianças que moram em bairros com menos dentistas têm maior incidência de cárie não tratada na dentição permanente.

## Introduction

It is well established that different individual factors influence the occurrence of dental caries in children and adolescents ^1^ ^,^ ^2^ ^,^ ^3^. However, studies have also demonstrated the influence of contextual factors, through the adoption of socioeconomic indicators of cities or neighborhoods and characteristics of the school context, on the development of dental caries ^2^ ^,^ ^3^ ^,^ ^4^. Social and structural aspects of neighborhoods, such as community support, collective cohesion, and number of entities in the community, including the number of workers and health service providers, have proven to be able to affect the health of residents ^5^ ^,^ ^6^ ^,^ ^7^.

The presence of associations and health centers within the neighborhood can facilitates access and demand for services by residents, the dissemination of good habits and also generates a positive environment, impacting health outcomes ^8^ ^,^ ^9^. Regarding to oral health, it is believed that the presence of dentists in the neighborhood can have a significant effect on oral health and the occurrence of dental caries among individuals, especially during the period from childhood to adolescence ^7^. This transition period is important and critical to health, due to the biological, cognitive, emotional, and social changes experienced by individuals, which can impact health and perpetuate throughout adult life.

To our knowledge, there are no studies that have assessed the influence of the number of dentists in the neighborhood on the incidence of dental caries during the transition period from childhood to adolescence. Thus, the longitudinal assessment of the influence of contextual factors in the neighborhood on normative oral health outcomes allows a broader understanding of the causal determinants of the health-disease process during an important period of biopsychosocial development. Therefore, the aim of this study is to evaluate the influence of the number of dentists in the neighborhood on the incidence of dental caries in permanent dentition. The conceptual hypothesis is that there will be a lower incidence of caries in children who live in neighborhoods with a higher number of dentists.

## Materials and methods

This study is reported according to STROBE (Strengthening the Reporting of Observational Studies in Epidemiology) guidelines ^10^.

### Ethical aspects

This study was approved by the Research Ethics Committee of the Federal University of Santa Maria (CAAE 54257216.1.0000.5346). Only children who agreed to participate and whose parents/guardians signed an informed consent form were included in this research.

### Study design and sample

The present study corresponds to a cohort with 7 years of follow-up in the city of Santa Maria, southern Brazil. The first stage (T1) took place in 2010, consisting of a systematic sample of 639 children aged 1 to 5 years. In 2010, the city had a population of approximately 261.031 citizens, among them, 27.520 were children under 6 years old ^11^. The sampling process included municipal health centers on the National Children’s Vaccination Day. The selected health centers were those with a dental chair (15 of 28 health centers), being distributed in different neighborhoods and covering all the 8 administrative regions of the city. Also, about 85% of children vaccinated that day were seen by the selected health centers. The complete methodology used for epidemiological research has been previously published ^12^.

The second follow-up reassessment (T2) took place 7 years later and data collection was conducted between January 2017 and March 2018. All 639 children participating in T1 were considered eligible and were invited to participate in the study. To locate these children, different search strategies were used: a) telephone calls; b) lists of students enrolled in public schools in the city. After having their addresses updated and the authorization of their parents/guardians, the localized children were reevaluated in a home or school environment by four previously trained and calibrated examiners. Demographic and socioeconomic variables were also collected, through a questionnaire answered by parents/guardians.

The sample size of this study was calculated considering a prevalence of dental caries of 47.2% in the non-exposed group (adolescents living in municipalities with a high median income) and 74.3% in the exposed group (adolescents living in municipalities with a low median income); standard error of 5%; confidence level of 95% (CI 95%); exposed/unexposed ratio 1:1; design effect of 1.6; and statistical power of 90% ^13^. Considering possible losses, 30% was added to the sample size. The minimum sample required was 309 children. As the present study is part of cohort that evaluates other conditions, a larger than required sample was included.

### Dental caries

At baseline and follow-up, the presence of dental caries was assessed through the number of Decayed, Missing, and Filled surfaces (DMF-S/dmf-s indexes) ^14^. At T1, the dental caries experience was considered as a proxy for broader risk factors in childhood that include excessive sugar consumption, lack of access to fluoride, low socioeconomic status, and so on ^4^ ^,^ ^7^. For data analysis, this variable was classified through the dmf-s ≥ 1, and categorized as “Without” or “With”. The incidence of untreated dental caries in permanent dentition (T2) was the study's outcome. In the baseline, no child had permanent teeth. Thus, all permanent teeth with untreated caries lesions at follow-up were considered teeth with new lesions (incidence). For the analysis, the number of surfaces with the presence of caries cavities (component D different from 0 in the DMF-S index) was considered.

The clinical examination was carried out by 4 examiners (graduate students) previously trained and calibrated for dental caries. The training and calibration process followed the criteria recommended by the World Health Organization for oral health research ^14^. Kappa values for intra-examiner and inter-examiner ranged from 0.72 to 0.95 and from 0.70 to 0.92, respectively. Oral examinations were performed at home or school with the aid of artificial light, periodontal probe (“ballpoint”), dental mirrors, and gauze.

### Number of dentists in the neighborhood (contextual variable)

The main predictive variable of the study was the presence of dentists in the neighborhood, which was collected at T1 through the number of existing dentists according to the geographic area (neighborhood) where the child was living, verified according to the official data published by the municipality. All professionals from the public and private network, with active registration in the Regional Dentistry Council (CRO) of the state, were considered, as conducted similarly in a previous study ^15^. For data analysis, this variable was used in a quantitative way. Characteristics related to social and structural aspects of neighborhoods have been previously used in the literature regarding health outcomes. The number of entities in the community, such as the number of workers and health service providers, have previously been tested and considered proxies of the structural characteristics of the neighborhood, such as social capital and income ^6^ ^,^ ^7^.

### Co-variables

Demographic and socioeconomic variables included gender (female and male), race, household income, and maternal education. Race was initially collected with the options considered by Brazilian Institute of Geography and Statistics (IBGE): white, black, brown, indigenous, or yellow, and after collection, it was dichotomized into “White” and “Non-white” ^11^. Household income was collected in *Reais* (official Brazilian currency - R$5.73 is equivalent to US$1.00, approximately) and considered all forms of income in the family. Subsequently, household income was dichotomized into “≥ 2 Brazilian minimum wages (BMW)” and “< 2 BMW” (2 BMW are equivalent to US$365.00). Maternal education was collected in terms of the number of years of schooling and was dichotomized in more or less than 8 years of formal education (incomplete primary education).

Behavioral and psychosocial variables included the dental attendance, the frequency of tooth brushing, and the parent’s perception of child’s oral health. The use of dental services was assessed by asking whether the children went to the dentist in the last 6 months. The reason for the visit was measured and categorized as follows: “Check-up/routine”; “Other reason”; “No visit”. The frequency of tooth brushing was recorded according to the number of times a day that the children brushed their teeth and was dichotomized in “≥ 2 times a day” and “< 2 times a day”. The perception of parents/guardians about child's oral health was collected through the question: “Would you say that the health of your child's teeth, lips, jaws and mouth is: 0 = Excellent; 1 = Very good; 2 = Good; 3 = Regular; or 4 = Bad. Further, the answers were dichotomized into: “Excellent/good” (score 0, 1 and 2) and “Regular/poor” (score 3 and 4).

### Statistical analysis

The program used for data analysis was STATA 14 (StataCorp. 2014. Stata Statistical Software: Release 14.1. College Station, TX: StataCorp LP). The descriptive analyzes of the sample were performed considering the sample weight, using the “svy” command for complex data samples in the STATA program. The comparison between followed and dropout children’s was assessed using the Chi-square test.

**Figure 1:**
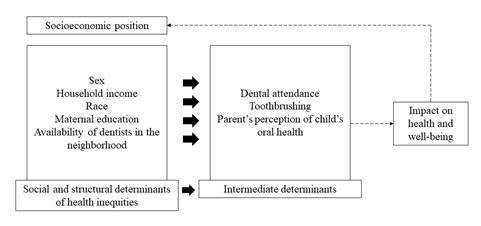
Adaptation of the model proposed by Solar and Irwin, using the study variables.

Multilevel Poisson regression was performed for the evaluation of the predictive variables in the incidence of untreated dental caries. The multilevel structure considered children (first level) nested in the 15 neighborhoods (second level). The variables were chosen based on the theoretical model proposed by WHO (Figure 1) and the variables that obtained p <0.20 in the unadjusted analysis, were included in the adjusted analysis ^16^. The results are presented in an incidence rate ratio (IRR) with the respective 95% confidence interval (95% CI). The analysis considered a fixed effect with a random intercept and the quality of the adjustment was assessed through deviance (-2 log likelihood) and median incidence rate ratio (MIRR).

The theoretical model proposed by the Commission on social determinants of health consists of two blocks ^15^. The first refers to the social and structural determinants of health inequities (such as context, education, income, and occupation); and the second, influenced by the first, covers intermediate determinants (such as housing, work, psychological factors, among others), which can influence the well-being and health of individuals ^16^.

## Results

A total of 639 children were assessed at T1 and 449 of them were reevaluated after 7 years of follow-up (cohort retention rate of 70.3%). The losses occurred due to the change of telephone or address (n = 181) or by the refusal of the parents/guardians (n = 9). The average age of participants in T1 and T2 was 2.8 years [standard deviation (SD): 1.4] and 10.0 years (SD: 1.4), respectively. Approximately, 63.0% of the children lived in neighborhoods with more than 1 dentist at T1 and 36.0% had at least one surface with untreated dental caries at T2.

Table 1 shows the children’s baseline characteristics and compares the group of children assessed with the group of children not assessed at T2. In the follow-up, the children were predominantly female, white, residing in households with a household income less than 2 BMW, and whose mothers had an education level (8 years. Also, most participants had not visited the dentist in the last 6 months, had a tooth brushing frequency of ≥ 2 times a day, did not experienced dental caries, and had their oral health assessed as good/excellent by their parents/guardians. Comparing the two groups, there was no statistically significant difference for all variables evaluated (p> 0.05).

**Table 1. Baseline:** characteristics (T1) between the group of children at baseline, who were followed up (T2) and who did not receive follow-up (T2).

Variables (T1)^a^	Baseline children (T1)^a^ (n = 639)	Followed up children (T2)^b^ (n = 449)	Dropout children (T2)^b^ (n = 190)	P-value*
	n (%)**	n (%)**	n (%)	
Sex				
Boys	322 (49.0)	220 (47.4)	102 (53.1)	0.28
Girls	317 (51.0)	229 (72.6)	88 (46.9)	
Race				
White	501 (80.5)	347 (79.6)	154 (82.7)	0.24
Non-white	137 (19.5)	102 (20.4)	35 (17.3)	
Maternal education				
( 8 years of formal education	357 (56.2)	246 (54.6)	111 (59.7)	0.35
< 8 years of formal education	275 (43.8)	199 (45.4)	76 (40.3)	
Household income (BMW)^c^				
≥ 2 BMW	212 (36.0)	144 (35.2)	68 (38.0)	0.26
< 2 BMW	390 (64.0)	282 (64.8)	108 (62.0)	
Dental attendance (last 6 months)				
Check-up/routine	94 (15.9)	63 (14.3)	31 (19.3)	0.64
Other reason	40 (6.4)	30 (7.3)	10 (4.5)	
No visit	496 (77.7)	349 (48.4)	147 (76.1)	
Toothbrushing				
≥ 2 times a day	444 (75.0)	321 (75.5)	123 (73.6)	0.35
< 2 times a day	148 (25.0)	101 (24.5)	47 (26.4)	
Dental caries experience				
Without	408 (63.3)	283 (63.6)	125 (62.7)	0.51
With	231 (36.7)	166 (36.4)	65 (37.3)	
Parent’s perception of child’s oral health				
Excelent/good	488 (76.8)	347 (76.8)	141 (76.7)	0.54
Regular/poor	149 (23.2)	102 (23.2)	47 (23.3)	
Number of dentists (mean [SE^d^])	11.6 (1.4)	15.7 (7.9)	17.1 (9.3)	0.70

*P-value of chi-square test; **Taking into account the sampling weight. Values lower than 639 at T1 and 449 at T2 due to missing data. ^a^T1: baseline.^b^T2: 7-year follow-up. ^c^BMW: Brazilian Minimal Wage (2 BMW was equivalent to US$365.00 approximately),^d^SE, standard error.

The sample distribution of the incidence of surfaces with untreated dental caries at follow-up (T2) according to the baseline characteristics (T1) are shown in Table 2. The mean of decayed surfaces at T2 was 0.92. Children who presented a higher incidence of surfaces with untreated caries in T2, were girls, not white, with maternal education lower than 8 years of formal education, household income < 2 BMW and who visited the dentist in the last 6 months for another reason (not routinely). Besides, children with dental caries experience, who showed a frequency of tooth brushing less than 2 times a day, whose parents perceived their oral health as fair/poor presented a higher incidence of surfaces with untreated dental caries. The number of dentists in the neighborhood was correlated with the mean of tooth with untreated dental caries (-0.10; p<0.05).

Table 3 shows the unadjusted and adjusted association between baseline characteristics (T1) and the incidence of surfaces with untreated dental caries (T2), determined by multilevel Poisson regression. After the adjustment, children who lived in neighborhoods with more dentists are more likely to present untreated dental caries (IRR 0.99; 95% CI 0.98-0.99). That is, with each increase in the mean number of dentists, there was a 1% reduction in the mean number of teeth with untreated dental caries. Children whose mothers had less than 8 years of schooling, who have not routinely visited the dentist in the last 6 months, and whose parents perceived their oral health as fair/poor showed a higher incidence of surfaces with untreated dental caries. Furthermore, children with dental caries experience also presented a higher risk of present untreated dental caries. The MIRR was 1.54 in the null model, 1.38 in the contextual model (number of dentists in the neighborhood), and 1.32 in the full model. The cluster variance (neighborhood) was significant in all models.

**Table 2:** Sample distribution of incidence of surfaces with untreated dental caries (T2) according to baseline characteristics (T1).

Variables (T1)^a^	Incidence of surfaces with untreated dental caries (T2)^b^
	Mean (SE)*
Sex	
Boys	0.83 (0.14)
Girls	1.01 (0.13)
Race	
White	0.88 (0.10)
Non-white	1.08 (0.25)
Maternal education	
( 8 years of formal education	0.71 (0.12)
< 8 years of formal education	1.19 (0.15)
Household income (BMW)^c^	
≥ 2 BMW	0.64 (0.12)
< 2 BMW	1.07 (0.13)
Dental attendance (last 6 months)	
Check-up/routine	0.68 (0.22)
Other reason	1.54 (0.52)
No visit	0.92 (0.11)
Toothbrushing	
≥ 2 times a day	0.93 (0.11)
< 2 times a day	0.87 (0.21)
Dental caries experience	
Without	0.74 (0.09)
With	1.42 (0.17)
Parent’s perception of child’s oral health	
Excelent/good	0.76 (0.10)
Regular/poor	1.47 (0.24)
Number of dentists	-0.10^d^

* SE, standard error (taking into account the sampling weight). ^a^T1: baseline. ^b^T2: 7-year follow-up. ^c^BMW: Brazilian Minimal Wage (2 BMW was equivalent to US$365.00 approximately). ^d^Pearson correlation.

**Table 3:** Unadjusted and adjusted association between baseline characteristics (T1) and incidence of surfaces with untreated dental caries (T2) determined using multilevel Poisson regression.

Incidence of surfaces with untreated dental caries				
Variables	IRR^a^ Unadjusted(95% CI)^b^	p-value	IRR^a^ Adjusted*(95% CI)^b^	p-value
Sex				
Boys	1	0.465	-	
Girls	1.07 (0.88-1.29)		-	
Race				
White	1	0.01	1	0.341
Non-white	1.34 (1.08-1.64)		1.11 (0.88-1.40)	
Maternal education				
≥ 8 years	1	<0.001	1	<0.05
< 8 years	1.60 (1.31-1.95)		1.28 (1.03-1.60)	
Household income (BMW)^c^				
≥ 2 BMW	1	<0.01	1	0.687
< 2 BMW	1.45 (1.15-1.83)		1.05 (0.82-1.35)	
Dental attendance (last 6 months)				
Check-up/routine	1	<0.01	1	<0.05
Other reason	2.73 (1.78-4.17)		1.78 (1.14-2.76)	
No visit	1.62 (1.16-2.27)		1.20 (0.85-1.68)	
Toothbrushing				
≥ 2 times a day	1	0.573	-	
< 2 times a day	0.93 (0.74-1.17)		-	
Dental caries experience				
Without	1	<0.001	1	<0.001
With	1.81 (1.49-2.20)		1.66 (1.32-2.08)	
Parent’s perception of child’s oral health				
Excelent/good	1	<0.001	1	<0.01
Regular/poor	2.01 (1.65-2.45)		1.36 (1.08-1.72)	
Number of dentists	0.99 (0.98-0.99)	<0.01	0.99 (0.98-0.99)	<0.01

a IRR, incidence rate ratio; ^b^CI, confidence interval; ^c^BMW: Brazilian Minimal Wage (2 BMW was equivalent to US$365.00 approximately). *Adjusted by dental caries in primary dentition at follow-up.

## Discussion

The present findings corroborate the hypothesis that children who live in neighborhoods with a higher number of dentists would have a lower incidence of dental caries throughout childhood. The results also suggest that individual, behavioral, and psychosocial variables are related to the development of the result. As far as we know, few longitudinal studies have assessed the influence of contextual variables on the occurrence of dental clinical outcomes and there are no studies evaluating the influence of the number of dentists in the neighborhood with the development of dental caries in children.

The presence of dentists in the neighborhoods positively influenced children's oral health. This scenario can be explained by several factors, including the relationship between the supply and use of health services. Previous studies have already indicated that the presence of associations and health centers in the neighborhood facilitates access and demand for services by residents, directly impacting health ^8^ ^,^ ^9^. It can be explained by the reason that users could be more likely to use services located in a family environment. Furthermore, the number of health professionals and service providers has already been associated with better health outcomes ^6^ ^,^ ^7^. Notwithstanding, it should be noted that the availability of dentists does not always mean effective access to dental services. A previous approach such as the “Inverse Care Law” emphasizes that both the amount of care available and the quality of care provided are inversely related to the need ^17^. Therefore, other explanations can also be used to explain the association between the number of dentists and dental caries.

In this context, the most number of dentists can also be related to the highest social capital in the neighborhood ^7^. Thus, communities with high social capital are characterized by the existence of associations and active citizens, which leads to a positive social environment, characterized by trust and social cohesion among resident individuals. Therefore, communities with several entities tend to have a larger social capital, to be more cohesive, and to disseminate good health habits. In this sense, collective social capital can influence health through psychosocial processes, behavioral paths, access to health services, and the development of public support policies ^18^. Therefore, societies with more dentists can provide better health for their residents, like fewer dental caries, as they have health-promoting characteristics. In this sense, it is recognized that oral health conditions are affected by the environment in which individuals are inserted, interpersonal relationships, and contextual factors.

About the association between caries experience at baseline and the increased risk of having untreated caries at follow-up, several predisposing factors for caries experience in child populations have been identified previously. Behavioral, demographic, socioeconomic, psychosocial and contextual determinants play a significant role in the development of caries disease ^1^ ^,^ ^4^. A previous study found that children evaluated for 7 years showed a significant difference in the disease trajectory, and the main predictor of caries in permanent dentition was the previous experience of caries ^19^. Considering children who were caries-free in the primary dentition and children with active caries are populations with different risk profiles, they need different prevention strategies ^19^. However, we emphasize that dental caries in the primary dentition was considered, in this study, as a proxy for dental caries in the permanent dentition, since the child remained with the disease most likely because of exposure to the same risk factors as in childhood.

Our findings demonstrated that the incidence of untreated dental caries was higher among children with mothers presenting less than 8 years of formal education. Individuals with higher educational levels tend to seek health services earlier and make better use of dental services, even in relation to services whose access is more difficult, such as specialized care ^7^ ^,^ ^20^. The mother education level can determine social opportunities, as well as healthy choices and restrictions, and influence children's oral health ^16^. Moreover, previous results confirmed that the mother's educational level was a statistically significant predictor for the increase in dental caries in children ^21^.

Children who have not been to the dentist or have not been routinely in the past 6 months had a higher incidence of untreated dental caries. A percentage of individuals who have never had a dental visit is still high and is associated with socioeconomic status ^22^. Previous studies pointed out that irregular or less frequent users of dental services had a lower number of dental restoration and a higher number of dental caries, when compared to regular users ^23^.

The regular or poor perception of the child's oral health by parents/guardians was also associated with a higher incidence of surfaces with untreated caries. The mother's perception of their child's oral health status has already been significantly associated with the clinical presentation of their child's dental caries ^24^. Studies have shown that mothers can accurately assess their children's oral health and that childrens of parents who are aware of their child's oral hygiene had a lower prevalence of dental caries and better oral hygiene ^24^ ^,^ ^25^ ^,^ ^26^. Thus, parents who positively perceive their child's oral health status are more likely to be aware of the cleaning of their teeth than those whose parents perceive otherwise ^24^. The positive perception of parents for their children's oral health was also associated with routine dental care, while the negative perception was associated with visits due a dental problems ^27^.

This study has some limitations, such as the absence of permanent teeth in the mouth at baseline. As the participants were between 1 and 5 years old (mean age = 2.8 years), the sample was in the primary dentition. However, this study aimed to verify the incidence of untreated dental caries in permanent dentition, assuming that at baseline there were no cases of dental caries in permanent teeth. Also, the external validity of the study may be affected, as 15 of the 28 health centers in the city were included in the data collection. Nonetheless, the selected centers covered around 85% of children participating in the vaccination program. In addition, the assessment of caries lesions at baseline and follow-up was conducted in different environments, which may have caused some bias. However, since that the DMFT examination does not depend on cleaning and drying, nor on artificial light to detect lesions activity, we believe that this fact did not interfere with our finding.

Another limitation is the use of the number of dentists as a proxy of structure, since that there are other variables that could be related to neighborhood structure, such as income, basic sanitation, political, and social cohesion ^3^ ^,^ ^4^ ^,^ ^8^ ^,^ ^16^. Furthermore, we accessed the number of dentists in the neighborhood according to CRO, not being able to assert that individuals used that dentist. However, this variable was used as a proxy for structural aspects of the neighborhood, such as income and social capital ^6^ ^,^ ^7^ ^,^ ^15^, reinforcing our findings. Additionally, the data present in the CRO's registration may be out of date and inaccurate. Nonetheless, this was the only official database available for consultation. Finally, the number of dentists in the neighborhood was assessed only at baseline and may have changed over 7 years, indicating caution in interpreting our results.

Despite the restrictions, this is a longitudinal study of 7 years of follow-up, with a high cohort retention rate (70.3%). Also, children were assessed at two different times in their lives, corresponding to early childhood at the beginning of the study and in the transition period to adolescence in T2. This longitudinal assessment of the influence of a contextual variable on the incidence of dental caries is especially important during the covered transition period, since it makes possible to acquire knowledge that can be applied in public health tools for this age group.

Our findings suggested that children who live in neighborhoods with fewer dentists have a higher incidence of untreated dental caries in permanent dentition. Since no previous study has assessed the influence of the number of dentists in the neighborhood on the incidence of caries in the transition from childhood to adolescence, this predictor could be a new contextual factor to help understand the causal determinants of caries disease in this transitional age group. Thus, the high number of dental surgeons in the neighborhoods can lead to a more structured and positive social environment and with greater social capital, which can influence the health-promoting among the residents and consequently contribute to reducing the incidence of dental caries in this population.
